# Low serum IGF1 is associated with hypertension and predicts early cardiovascular events in women with rheumatoid arthritis

**DOI:** 10.1186/s12916-019-1374-x

**Published:** 2019-07-22

**Authors:** Malin C. Erlandsson, Lovisa Lyngfelt, N. David Åberg, Caroline Wasén, Rachelle A. Espino, Sofia Töyrä Silfverswärd, Mitra Nadali, Katharina Jood, Karin M.E. Andersson, Rille Pullerits, Maria I. Bokarewa

**Affiliations:** 1 Department of Rheumatology and Inflammation Research, Institute of Medicine, the Sahlgrenska Academy at University of Gothenburg, Guldhedsgatan 10A, SE-41345 Gothenburg, Sweden; 2 Rheumatology Clinic, the Sahlgrenska University Hospital, Gothenburg, Region of West Götaland Sweden; 3 Department of Internal Medicine, Institute of Medicine, the Sahlgrenska Academy at University of Gothenburg, Gothenburg, Sweden; 4 Keele University, Keele, UK; 5 Department of Clinical Neuroscience, Institute of Neuroscience and Physiology, the Sahlgrenska Academy at University of Gothenburg, Gothenburg, Sweden; 6 Department of Neurology, the Sahlgrenska University Hospital, Gothenburg, Sweden; 7 Department of Clinical Immunology and Transfusion Medicine, the Sahlgrenska University Hospital, Gothenburg, Sweden

**Keywords:** IGF1, Ischemic stroke, Hypertension, Rheumatoid arthritis, Inflammation

## Abstract

**Objectives:**

Since low insulin-like growth factor (IGF) 1 is often linked to inflammation, we analyze whether serum levels of IGF1 are associated with cardiovascular disease (CVD) in rheumatoid arthritis (RA) in a longitudinal observational study.

**Methods:**

A CVD risk was estimated (eCVR) in 184 female RA patients (mean age 52 years) and in 132 female patients after ischemic stroke (mean age 56 years) with no rheumatic disease, using the Framingham algorithm. The median level of IGF1 divided the cohorts in IGF1^high^ and IGF1^low^ groups. A 5-year prospective follow-up for new CVD events was completed in all RA patients. The Mantel-Cox analysis and event-free survival curves were prepared. Unsupervised clustering of proteins within the IGF1 signaling pathway was employed to identify their association with eCVR.

**Results:**

Low IGF1 resulted in a higher eCVR in RA patients (7.2% and 3.3%, *p* = 0.0063) and in stroke (9.3% and 7.1%, *p* = 0.033). RA had higher rate for new CVD events at prospective follow-up (OR 4.96, *p* = 0.028). Hypertension was the major risk factor associated with low IGF1 in RA and stroke. In hypertension, IGF1 was no longer responsible for intracellular activation and lost its correlation to IRS1/2 adaptor proteins. The clustering analysis confirmed that combination of low IGF1 and IRS1/2 with high IL6, insulin, and glucose predisposed to high eCVR and emphasized the functional role of serum IGF1.

**Conclusions:**

Low serum IGF1 precedes and predicts development of early CVD events in female RA patients. Hypertension and aberrant IGF1 receptor signaling are highlighted as the important contributors to IGF1-related CVD events.

## Background

Hypertension is a significant health threat and an independent predictor of CV events including coronary heart disease, stroke, heart failure, and dementia in the general population [1] and in RA [2]. The prevalence of hypertension in rheumatoid arthritis (RA) is high, but varies between 4 and 72% producing inconsistent results in the repeated meta-analysis [3–5]. Studies in RA population often indicated high rates of hypertension in patients with first stroke [6–9] and the highest stroke risk in young patients < 50 years [10]. Chronic inflammation, the hallmark of RA and an important contributor to the high CVD rate in these patients, is viewed as a major pathogenic process behind significant vascular dysfunction. Inflammation reduces artery elasticity and increases systemic vascular resistance, which consequently leads to an increased arterial blood pressure (BP) [11–13]. Elevated C-reactive protein levels are associated with hypertension in the general population by reducing the production of endothelial nitric oxide and by triggering platelet activation and leukocyte adhesion. Pro-inflammatory cytokines TNF-a and IL6 maintain hypertension by inducing endothelial proliferation and increasing vascular permeability and blood volume [14, 15].

Solid biological evidence connects insulin-like growth factor 1 (IGF1) with the regulation of endothelial cell function. Vascular endothelial and smooth muscle cells express IGF1 receptor (IGF1R), which mediates angio-protective effects of IGF1. Locally produced IGF1 supports proliferation and migration of endothelial progenitors essential for blood vessel reparation and controls oxidative stress triggered by inflammation [16–18]. Circulating IGF1 is known to induce vasodilation, which contributes to the regulation of arterial BP and vascular tone, while deletion of IGF1 in mice enhanced mechanisms of vasoconstriction leading to hypertension. Clinically, both a direct and inverse relation between IGF1 levels and BP has been reported. Early studies with focus on patients with congenital or acquired abnormalities in GH/IGF1 production, indicated an association between high IGF1 production and hypertension [19, 20]. Recent studies of general population with low prevalence of obvious endocrinological conditions reported an inverse association between IGF1 and BP [21–24]. Low serum IGF1 is tightly linked to vascular changes in aging [25] and obesity [26] and to the increased cardiovascular morbidity and mortality [27–30].

Disturbances in IGF1/IGF1R signaling are notable for RA pathology. It contributes to joint inflammation and affects homeostasis in chondrocytes, leukocytes and synovial fibroblasts [31–33]. It has been shown that inflammation is directly related to high expression of IGF1R in leukocytes [34], which might be responsible for higher pain perception in RA [33, 35]. In a study combining two independent RA cohorts, we demonstrated that smoking and inflammation had additive suppressive effects on serum IGF1 levels [36]. These reports imply IGF1/IGF1R signaling to be a natural protective response to inflammation and pain. Little is known whether IGF1 contributes to the prevalence of CVD in RA. Therefore, the objective of this study was to determine the importance of low IGF1 and alterations in IGF1/IGF1R signaling for CVD in RA and to assess whether similarities exist in patients without RA afflicted by ischemic stroke.

## Methods

### The RA cohort

This study involved 184 female RA patients consecutively enrolled at the Rheumatology Clinic of Sahlgrenska University Hospital, Gothenburg, and the Northern Älvsborg County Hospital, Uddevalla [33, 36]. All the patients fulfilled the classification criteria for RA (ACR1987) and had median disease duration of 7 years. The dominating majority (89%) were RF and/or ACPA positive. Active anti-rheumatic treatment had 175 patients. Among those, monotherapy with MTX had 82 patients (47%), other DMARDs 3 patients (1.7%); combination therapy with DMARDS 45 patients (26%), TNF-a-inhibitors 30 patients (17%), and other biologics 15 patients (8.6%). The study is registered at the Clinical Trials.gov with ID NCT03449589.

### The ischemic stroke (IS) cohort

We use clinical and serological data collected at 3 months after index IS of 132 consecutive female patients recruited at the Sahlgrenska University Hospital in the frame of the Sahlgrenska Academy Study on Ischemic Stroke (SALHSIS) [30, 37].

The studies are approved by the Swedish Ethical Review Authority (RA cohort, Dnr.659-11 and SALHSIS cohort, Dnr.Ö469-99) and are conducted in accordance with the ethical principles of the Helsinki Declaration and Good Clinical Practice. All patients provided written informed consent before enrolment into the study.

### Cardiovascular risk

A 10-year risk of developing CVD was estimated (eCVR) using a digital version of the Framingham algorithm [38] and included sex, age, systolic BP, treatment for hypertension, current smoking, diabetes mellitus, HDL, and TC.

### Prospective follow-up

Five years after enrollment the RA patients were contacted for a structured telephone interview. The questions were asked for any CVD events, cases of type 2 diabetes (T2D) and current medication including anti-hypertensive drugs, anticoagulants, anti-diabetic drugs, and statins. The reported CVD events and changes in medications were then controlled against medical records and the Swedish National Patient Registry.

*Hypertension* was recorded if an incidental measure of systolic BP was > 140 mmHg, or diastolic BP was > 90 mmHg or pharmacological treatment for hypertension was used. The BP burden was calculated as a sum of systolic and diastolic BPs.

*Disease activity score (DAS28) of RA* was calculated based on assessment of 28 tender and swollen joints and ESR.

### Blood sampling and storage

The samples were collected between 7 and 10 o’clock in the morning after overnight fasting. For serum preparation, the blood was obtained from the cubital vein into vacuum containers (BD Vacutainer) and for RNA preparation into PAXgene protection tubes (Becton Dickinson, Franklin Lakes, NJ, USA). Serum samples were stored at − 70 °C and PAXtubes in − 20 °C until use.

### Serological measurements

In RA samples, serum IGF1, total cholesterol (TC), triglycerides, high-density lipoprotein (HDL), and low-density lipoprotein (LDL) were measured by photometry on Cobas 8000 (Roche Diagnostics, Switzerland). In the IS samples, serum IGF1 was measured with a radio-immune assay (Mediagnost, Reutlingen, Germany) [39]. Plasma glucose levels were measured using FreeStyle Lite (Abbott Diabetes Care Ltd., Oxon, UK). Sandwich ELISAs were used to measure insulin (DY8056, R&D Systems, Minneapolis, MN, USA) and IL6 and IL1b (M9316 and M1934, respectively; Sanquin, Amsterdam, the Netherlands).

### Gene expression analysis

Total mRNA was prepared using PAXgene Blood RNA kit (Qiagen). Complementary DNA was synthesized using High Capacity cDNA Reverse Transcription kit (Applied Biosystems, Foster City, CA). Amplification of the gene product was attained on a ViiA™7 Real-Time PCR (Applied Biosystems) using SYBR Green qPCR Mastermix (SA Biosciences, Qiagen) and primer pairs as reported [33, 40]. Gene expression levels were calculated by the ddCt method and presented as relative quantity to the average expression in the IGF1^hi^ group.

### Statistical analysis

The SPSS v.25 (IBMSPSS, Chicago, IL), GraphPad prism v.7, www.open-epi.com, R v.3.3.0 (R Core Team, 2018) and R studio v.1.1.447 (http://www.rstudio.com/) were used for the analysis. Data are presented as mean ± SD, median [IQR], or in absolute numbers. Missing data for BP (5%) and ESR (8%) were imputed using the linear regression (SPSS). The study cohorts were dichotomized into IGF1^hi^ and IGF1^low^ groups by the median level. Continuous data were analyzed using the Mann-Whitney *U* test, the Kruskal-Wallis test followed by Dunn’s post hoc test, and Spearman’s correlation test. Relative risk prediction was done using the area under the receiver operative characteristic (ROC) curve. The Kaplan-Meier curves and the Mantel-Cox analysis were used to compare the groups. For the clustering analysis, the data were log normalized and ranked by row. Heatmaps and hierarchical clustering were performed in R, using the stats and gplots packages, Spearman correlation-based distances, and Ward2 linkage. All tests were two-tailed and conducted with 95% confidence.

## Results

### Low IGF1 levels are associated with higher CVD risk in RA patients

**Fig. 1:**
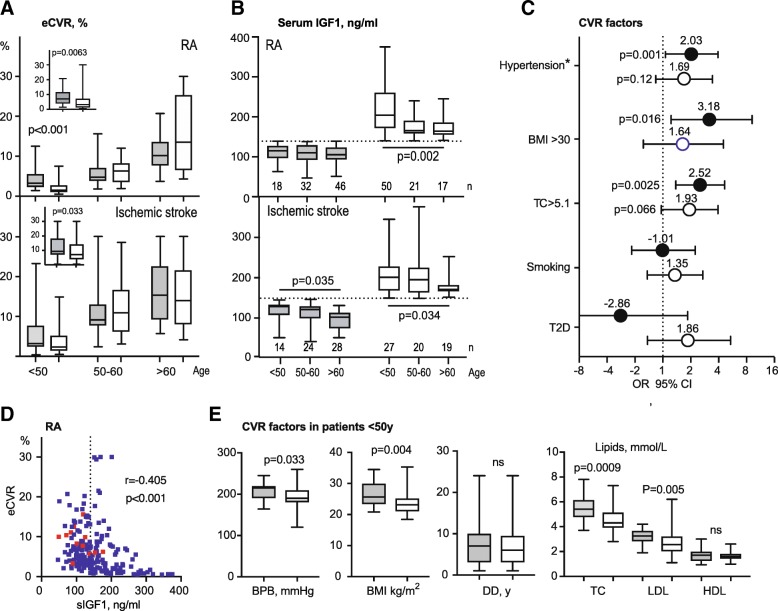
Low serum levels of IGF1 are associated with higher estimated cardiovascular risk (eCVR) in RA patients. **a** eCVR was calculated in 184 female RA patients and in 132 female incidental ischemic stroke using the Framingham lipid algorithm. The median level of IGF1 formed the IGF1high and IGF1low groups. Box plots show eCVR separately for IGF1^low^ and IGF1^hi^ groups stratified by age. Embedded box plots show eCVR in the total cohorts. **b** Box plots show levels of IGF1 separately for IGF1^low^ and IGF1^hi^ groups stratified by age. Box plots present median, interquartile range. *P* values are calculated with the Mann-Whitney *U* test. **c** The forest plot shows the difference in traditional CVR factors between IGF1^low^ and IGF1^hi^ groups is shown as odds ratio (OR) with 95% confidence interval (CI). Filled circles indicate RA cohort (*n* = 184), open circles Ischemic stroke cohort (*n* = 132). The *p* values are obtained by chi-square statistics. BMI, body mass index; TC, total cholesterol; T2D, type 2 diabetes. **d** The Spearman correlation between eCVR and serum levels of IGF1 in the RA cohort is shown as a dot plot. The dotted line indicates the median IGF1 level separating IGF1^low^ and IGF1^hi^ groups. Red dots indicate the patients, who developed CV events during the prospective follow-up. **e** The comparison of CVR factors between IGF1^low^ (*n* = 18) and IGF1^hi^ (*n* = 50) patients within the youngest age group < 50 years. Box plots present median, interquartile range. P-values are calculated with the Mann-Whitney *U* test. BPB, blood pressure burden (a sum of systolic and diastolic BP); BMI, body mass index; DD, disease duration; TC, total cholesterol; LDL, low-density lipoproteins; HDL, high-density lipoproteins

Consistent with a clinically relevant IGF1-deficiency, the IGF1^low^ group was lower in height compared to IGF1^hi^ group (Table 1). The IGF1^low^ group had significantly higher eCVR compared to the IGF1^hi^ group (7.0% vs. 3.2%, *p* = 0.0063), and the absolute IGF1 levels correlated negatively to the eCVR (Fig. 1a, b, d). Since both eCVR and IGF1 levels were age dependent, the analysis was performed separately within patients of different age. Expectedly, the eCVR had a gradual increase with age, both in the IGF1^low^ and IGF1^hi^ groups (Fig. 1b). The increase in eCVR was predominantly attributed to IGF1^low^ patients < 50 years. Notably, these patients were associated with significantly higher BP burden, BMI, TC, and LDL, compared to the age-matched IGF1^hi^ group (Fig. 1d). Notably, the IGF1^low^ RA patients < 50 years reached a low threshold of IGF1 levels with no further decline (Fig. 1b). This low IGF1 was associated with significantly higher BP burden, BMI, TC, and LDL, compared to the age-matched IGF1^hi^ group (Fig. 1e).

**Table 1:** Clinical characteristics of female RA and stroke cohorts

	RA		Ischemic stroke	
	Low IGF1 < 140 ng/ml	High IGF1	Low IGF1 < 145 ng/ml	High IGF1
*n*	96	88	66	66
Age, years	59.0 [51.2–63]	47.5 ^*p* < 0.0001^ [34.5–55.3]	58.5 ^*p* = 0.73^ [51–66]	52.7 ^*p* = 0.004^ [39.8–61.7]
Serum IGF1, ng/ml	109.5 [93–127]	182.5 ^*p* < 0.0001^ [158–225]	111.6 ^*p* = 0.74^ [92–127]	183 ^*p* < 0.0001^ [167–223]
Height, cm	166 [161–169]	167 ^*p* = 0.041^ [163–172]	163 ^*p* = 0.005^ [160–167]	167 ^*p* = 0.009^ [161–172]
Systolic BP, mmHg	135 [120–145]	121 ^*p* = 0.0027^ [120–140]	135 ^*p* = 0.70^ [120–140]	130 ^*p* = 0.114^[120–140]
Diastolic BP, mmHg	80 [75–85]	80 ^*p* = 0.023^ [70–80]	80 ^*p* = 0.037^ [70–80]	75 ^*p* = 0.058^ [70–80]
TC, mmol/L	5.6 [4.9–6.0]	5.0 ^*p* = 0.0022^ [4.1–5.9]	5.1 ^*p* = 0.0004^ [4.5–5.8]	4.8 ^*p* = 0.063^ [4.3–5.5]
LDL, mmol/L	3.4 [2.8–4.0]	3.0 ^*p* = 0.17^ [2.3–3.8]	2.8 ^*p* < 0.0001^ [2.1–3.50]	2.6 ^*p* = 0.89^ [2.30–3.30]
HDL, mmol/L	1.8 [1.5–2.2]	1.7 ^*p* = 0.20^ [1.5–2.1]	1.6 ^*p* = 0.0002^ [1.30–1.90]	1.5 ^*p* = 0.13^ [1.10–1.80]
BMI, kg/m^2^	25.9 [23.1–29.7]	23.9 ^*p* = 0.0014^ [21.9–26.7]	24.0 ^*p* = 0.17^ [21.8–28.3]	24.6 ^*p* = 0.94^ [22.5–28.0]
T2D, *n* (%)	2 (2.1%)	4 ^*p* = 0.39^ (4.5%)	12 ^*p* = 0.0004^ (19%)	7 ^*p* = 0.23^ (9%)
Current smokers, *n* (%)	15 (16%)	13 ^*p* = 0.88^ (16%)	32 ^*p* < 0.001^ (48%)	28 ^*p* = 0.49^ (45%)
Hypertension*, *n* (%)	25 (26%)	7 ^*p* = 0.011^ (8%)	30 ^*p* = 0.012^ (45%)	28 ^*p* = 0.17^ (42%)

*IGF1* insulin-like growth factor 1, *RA* rheumatoid arthritis, *DAS28* Disease Activity Score with assessment of 28 joints, *TC* total cholesterol, *HDL* high-density lipoprotein, *LDL* low-density lipoprotein, *BMI* body mass index, *T2D* type 2 diabetes, *CVR* cardiovascular risk

Median [IQR], group comparison was done by unpaired *t* test statistics and proportions by chi-square. *p* values in the IGF1^hi^ column indicate the differences between the IGF1^hi^ and IGF1^low^ groups; *p* values in the IGF1^low^ column indicate the differences between the IGF1^low^ groups of RA and post-stroke patients

*Hypertension is defined as systolic BP ≥ 140 mmHg and/or diastolic BP ≥ 90 mmHg

The comparison of the traditional CVR factors between IGF1^low^ and IGF1^hi^ groups showed that hypertension was the most prominent distinctive feature (Fig. 1c). IGF1^low^ group had higher rate of pharmacological treatment for hypertension. The incidental measures of systolic and diastolic BP in IGF1^low^ groups were also higher compared to IGF1^hi^ (Table 1). Hypercholesterolemia and higher levels of BMI, LDL, and HDL were also characteristic for the IGF1^low^ group, while there was no association with diabetes or smoking (Fig. 1c, Table 1).

### Low IGF1 levels and CVD risk in IS patients

Age and serum IGF1 levels in the IS cohort were comparable with RA patients (Table 1, Fig. 1b). The IGF1^low^ IS patients had higher diastolic BP and eCVR (9.3% vs. 7.0%, *p* = 0.033) (Fig. 1a). However, there was no significant difference between IGF1^low^ and IGF1^hi^ IS groups with respect to the individual CVR factors (Table 1, Fig. 1c). Compared to the RA cohort, the rate of hypertension, smoking, and diabetes was higher in the IS cohort, whereas levels of TC, HDL, and LDL were lower. Pharmacological treatment of hypertension and hyperlipidemia, which is prescribed actively during the early post-stroke phase, will probably explain this difference.

### CVD risk in relation to RA disease and inflammation

**Fig. 2:**
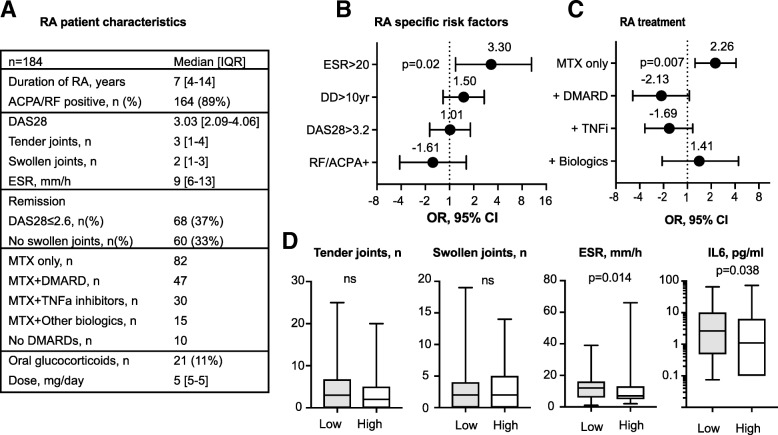
Cardiovascular risk factors attributed to rheumatoid arthritis. **a** The table shows patient characteristics of the RA cohort. Forest plots show the difference between IGF1^low^ (*n* = 96) and IGF1^hi^ (*n* = 88) groups in RA-specific parameters (**b**) and in anti-rheumatic treatment (**c**). Lines indicate odds ratio (OR) with 95% confidence interval (CI). The p-values are obtained by chi-square statistics. **d** The comparison of clinical and serological parameters between IGF1^low^ (filled boxes) and IGF1^hi^ (open boxes) groups. Box plots show median, interquartile range. P-values are calculated with the Mann-Whitney U test. RA, rheumatoid arthritis; ESR, erythrocyte sedimentation rate; DD, disease duration, DAS28, disease activity score with assessment of 28 joints; RF, rheumatoid factor; ACPA, antibodies to cyclic citrullinated peptides; MTX, methotrexate; DMARDs, disease modifying anti-rheumatic drugs; TNFi, tumor necrosis factor inhibitors

Clinical characteristics of RA patients are shown in Fig. 2a. Analysis of the RA-related CVR including RA-specific antibodies, active RA with DAS28 ≥ 3.2, and disease duration > 10 years, identified no differences between the IGF1^low^ and IGF1^hi^ groups (Fig. 2b). Despite the absence of obvious differences in clinical disease activity and similar numbers of swollen and tender joints, IGF1^low^ group had the higher frequency and absolute measures of ESR and IL6 (Fig. 2b, d). This led us to ask if IGF1^low^ and IGF1^hi^ patients needed a similar amount of treatment to control the RA disease. To investigate this, we compared the frequency of MTX monotherapy and combination therapy in the groups (Fig. 2c). The IGF1^low^ group was significantly more often treated with MTX monotherapy (OR 2.26, *p* = 0.007), and the dose of MTX was similar to IGF1^hi^ group (18.7 vs. 15.0 mg/week, ns), which implied insufficient inflammation control. The combination of MTX with other DMARDs or TNF-a inhibitors was not prevalent in the IGF1^hi^ group (Fig. 2c).

### Low IGF1 associates with new CVD events

**Fig. 3:**
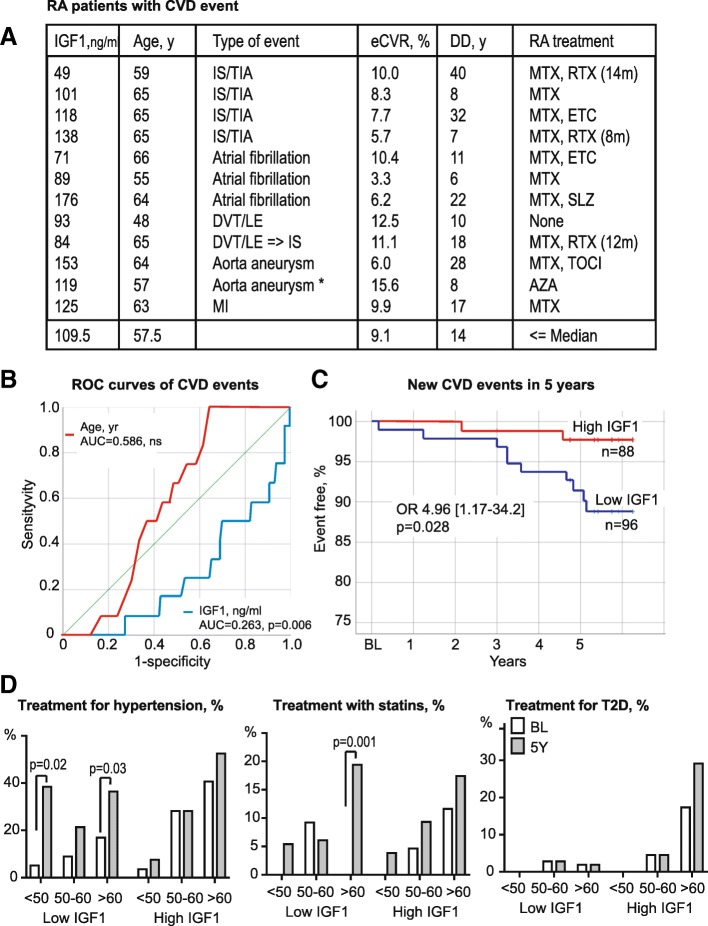
Predictive performance of serum IGF1 levels for development of cardiovascular events in rheumatoid arthritis. **a** The table shows individual baseline characteristics of 12 patients, who developed CV events during the 5-year follow-up. Asterisk indicates the case of death. **b** The receiver operator characteristic (ROC) curves depict associations between CV events developed during the 5-year follow-up and age (red line) and absolute serum levels of IGF1 (blue line) at baseline in 184 female RA patients. AUC, area under the ROC curve. **c** The Kaplan-Meier curves show the development of new CV events in IGF1^hi^ (*n* = 88, red line) or IGF1^low^ (*n* = 96, blue line) groups. **d** The column diagrams show the frequency of treatment at baseline (BL, open columns) and at the 5-year follow-up (5Y, filled columns) in IGF1^low^ and IGF1^hi^ groups stratified by age, < 50 years (*n* = 68), 50–60 years (*n* = 53), > 60 years (*n* = 63). The *p* values are obtained by chi-square statistics. eCVR, CV risk estimated by the Framingham algorithm; DD, disease duration; IS, ischemic stroke; TIA, transitory ischemic attack; DVT, deep venous thrombosis; LE, lung embolism; MI, myocardial infarction; MTX, methotrexate, RTX, rituximab; ETC, etanercept; SLZ, sulfasalazine; AZA, azathioprine; TOCI, tocilizumab; OR, odds ratio; CI, confidence interval; T2D, type 2 diabetes

At the 5-year follow-up, new CVD events occurred in 12 of 184 RA women (6.5%). The baseline characteristics of patients with new CVD events are shown in Fig. 3a. We learned that new CVD events often belong to established complications of hypertension and include 4 cases of ischemic stroke, 3 atrial fibrillations, and 2 aorta aneurysms. Patients with new CVD events had longer disease (14 vs. 7 years, *p* = 0.006) than the average of the RA cohort. They were slightly older (57.5 vs. 53 years, not significant) and had the eCVR of 9.1%, similar to the IS cohort.

To assess the clinical importance of serum IGF1 for development of new CVD events, we performed the ROC analysis of new CVD events using the absolute levels of IGF1 and age as independent variables. The ROC curve identified a strong negative relation between IGF1 and CVD events (Fig. 3b) and confirmed higher eCVR in IGF1^low^ group (Fig. 1a). The ROC curve with age showed no association to CVD events and confirmed independent of age input of IGF1 to the developed CVD events. Indeed, the absolute CVD risk in IGF1^hi^ group was 2.3% and in IGF1^low^ group, it was 10.4% giving the relative risk of CVD event 3.47 times higher for the patients in the IGF1^low^ group. To assess the probability of future CVD events, we constructed the event-free survival curves for IGF1^low^ and IGF1^hi^ groups (Fig. 3c). The Mantel-Cox analysis of the curves revealed an almost 5 times higher probability of CVD event in IGF1^low^ group (OR 4.96, *p* = 0.028).

During the 5-year period, we observed a remarkable increase in treatment for hypertension to occur in IGF1^low^ but not in the IGF1^hi^ group (Fig. 3d). This increase was significant among patients < 50 years and ≥ 60 years, which could have partly limited development of new CVD events in young RA patients.

### Hypertension is associated with an imbalance in the signaling through IGF1 receptor

**Fig. 4:**
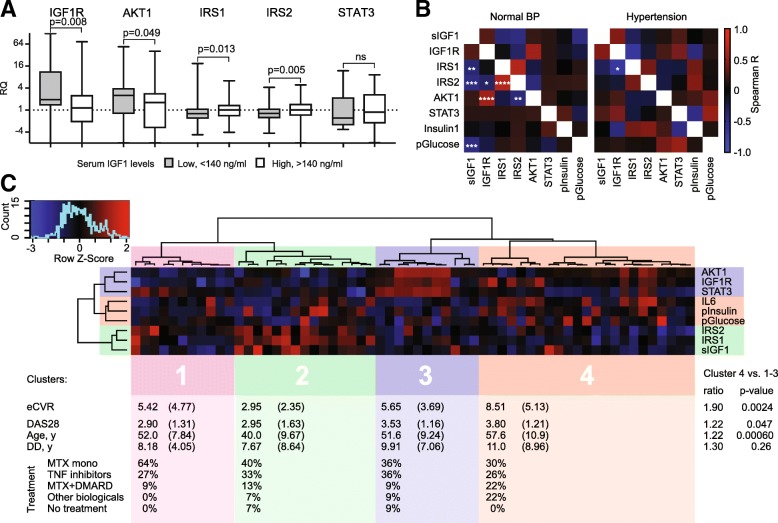
The IGF1 signaling pathway is related to hypertension and cardiovascular risk. **a** Box plots show mRNA levels in leukocytes of patients within IGF1^hi^ or IGF1^low^ groups. **b** Correlation matrix shows Spearman’s R values in color, as indicated the color key code, in patients with normal blood pressure (BP) or hypertension. *p < 0.05, **p < 0.01, ***p < 0.001, ****p < 0.0001. **c** Heatmap shows individual components related to IGF1 signaling of 60 RA patients. Unsupervised clustering analysis was done using the Spearman’s correlation-based distances and Ward2 linkage and formed 4 clusters. The table represents the mean and standard deviation of the parameters by cluster. The difference between clusters 1–3 and 4 was calculated with the t-test. RQ, relative quantity; MTX, methotrexate; TNFi, tumor necrosis factor inhibitors; DMARD, disease modifying anti-rheumatic drugs; DAS28, disease activity score with an assessment of 28 joints; DD, disease duration

To assess how low serum IGF1 affects IGF1R signaling in leukocytes, we compared the transcription of individual proteins within this pathway between IGF1^low^ and IGF1^hi^ groups in RA. We observed that IGF1^low^ group had a significantly higher mRNA of IGF1R and AKT1, and low mRNA of the adaptor proteins IRS1 and IRS2 (Fig. 4a). To study how these changes are related to hypertension, the clinical hallmark of low IGF1, we compared internal correlations between the individual components in normotensive and hypertensive patients (Fig. 4b). We observed that the negative correlations between IGF1 and IRS1, IRS2 and plasma glucose, which were present in the normotensive patients, could no longer be found in the hypertensive patients. Instead, novel correlations between IGF1R, IRS1, IRS2, and insulin appeared with hypertension demonstrating that IGF1 is no longer responsible for activation of intracellular processes through IGF1R. Finally, we performed an unsupervised clustering of RA patients by individual components of the IGF1 signaling pathway aiming to identify associations with the increased eCVR. Unsupervised clustering analysis of 60 patients with the complete set of the individual components formed four independent clusters (Fig. 4c). At visual inspection, cluster 4 had highest eCVR (mean 8.51) combined with lower IGF1, IRS1, and IRS2, while serum IL6, insulin, and glucose was high. This cluster differed significantly from the other clusters by eCVR (*p* = 0.0024), DAS28 (*p* = 0.047) and age (*p* = 0.0006), but had similarities in eCVR with patients developed new CVD events (Fig. 3a). Cluster 2, opposite to cluster 4, had the lowest eCVR, high levels of IGF1 and IRS1/2 and low IL6, insulin, and glucose. Clusters 1 and 3 had intermediate eCVR and low levels of IGF1, IRS1/2, IL6, insulin, and glucose (Fig. 4c).

## Discussion

In this cross-sectional study, we explored longitudinal associations between serum IGF1 and early CVD in RA. We showed harmful consequences of normal-low levels of IGF1 for the increase in the eCVR in female RA patients < 50 years and premature development of new CVD events. The profile of new CVD events consisted of the established complications of hypertension including ischemic stroke, atrial fibrillation, and aorta aneurysm, which indicated that hypertension was an essential clinical sign of IGF1-related CVD of the studied patients. Indeed, the patients with low IGF1 demonstrated higher BP measures at baseline and a significant increase in treatment for hypertension at the prospective follow-up. These findings in RA patients are consolidated by the reference cohort of IS females of similar age, where a significant association between low IGF1, hypertension, and eCVR is also observed.

The cumulative knowledge gained in a recent meta-analysis supports a direct causality between low IGF1 and hypertension [22]. Although the studied cohorts had no patients with extreme levels of IGF1, we observed a pronounced negative effect of normal-low IGF1 levels on the development of CVD and on its arterial localization. Our findings offer an appropriate molecular context to the high rate of stroke previously reported in young RA patients [10]. We found that the IGF1-related increase in eCVR was most noticeable in young women < 50 years. This implies that the vascular effects related to IGF1 may precede severe atherosclerotic change in the vessel wall and predispose to its early development. Indeed, previous studies in RA connected early CV events with endothelial dysfunction [11, 12]. Direct angioprotective properties of IGF1 for vascular endothelial cell function have been extensively studied and is experimentally verified [16]. In clinical studies, substitution with IGF1 was often followed by resolution of hypertension and improvement of the intima-media thickness of the arterial vessel wall [41–43]. Importantly, IGF1 has an immediate effect on repairing tissue damage after acute ischemic cerebral and myocardial injury [39, 44, 45]. In RA, the factors enhancing IGF1 levels attracted less attention. In this study, we observed that the use of TNF-a inhibitors was associated with higher IGF1 levels, while patients with low IGF1 received mostly MTX monotherapy. Treatment with TNF-a inhibitors is suggested to improve hypertension through an endothelium-dependent mechanism [46, 47], which could indicate a contribution of increasing IGF1 levels. We would expect that anti-rheumatic treatment assisting production and bioavailability of IGF1 to be an important tool to recover cardiovascular health in RA patients. Physical inactivity, a condition accumulated in long-lasting RA and in ischemic stroke [8], is frequently associated with lower serum IGF1 [48] that could be increased with physical exercise [49]. Changing lifestyle to improve serum IGF1 levels could be an attractive alternative for RA patients [50].

In the present study, we investigated how the difference in serum IGF1 changed the IGF1R signaling and its relation to eCVR. From analyzing leukocytes, we confirmed that serum IGF1 controlled the relative expression of proteins within the IGF1R pathway. Importantly, the relation between these proteins changes with hypertension, demonstrating its intimate connection with low IGF1. The unsupervised clustering of the IGF1-related proteins proposed the high CVR signature, which combined the low expression of IGF1, IRS1, and IRS2 with high serum IL6, insulin and plasma glucose and emphasized the functional role of serum IGF1 in the development of early CVD in RA. These findings are in line with our recent report in experimental arthritis, which puts forward the role of excessive inhibitory phosphorylation in IRS1 to create a condition of insulin/IGF1 resistance [35]. The situation seems more complex in patients, where low IGF1 is concurrent with high IGF1R and low adaptors IRS1/2. Being an ancestor of pro-insulin, IGF1 modulates carbohydrate metabolism, which stimulates glucose transport and inhibits insulin sensitivity [18]. In the general population and experimentally, IGF1 deficiency leads to glucose intolerance and T2D, the conditions rare among the studied RA patients. This was despite the fact that metabolic deviations as obesity, hyperlipidemia and hyperinsulinemia were expectedly frequent in IGF1^low^ group.

There are notable strengths and limitations in our study. Among strengths, this is to our knowledge the largest study of the relation between IGF1 levels and cardiovascular morbidity in RA, and the only one to explore the longitudinal association with the development of CVD events. An additional strength is the similarity of the RA and IS female cohorts, which comprise women of the same age. This study is done on the female cohort of RA patients and allows no extrapolation of the results on male RA patients. Additionally, the study is restricted to the risk of CV events and does not cover cerebrovascular health in female RA patients, since the vascular wall has not been evaluated. Hence, it is outlined among the limitations of our study. We believe that the chosen methodology does not affect the results obtained in the between-group comparison.

## Conclusion

Taken together, the results of this study attract attention to serum levels of IGF1 as a valuable parameter for estimating CVD risk in female RA patients. Low serum IGF1 precedes and predicts the development of early CVD events due to a tight connection with hypertension, which is important for future management of RA patients.

## Data Availability

The datasets used and/or analyzed during the current study are available from the corresponding author on reasonable request.

## Abbreviations

ACPA: Anti-citrullinated protein antibodies
AZA: Azathioprine
BMI: Body mass index
BP: Blood pressure
BPB: Blood pressure burden
CI: Confidence interval
CVD: Cardiovascular disease
CVR: Cardiovascular risk
DAS28: Disease activity score based on 28 joints and ESR
DD: Disease duration
DMARD: Disease modifying anti-rheumatic drugs
DVT: Deep venous thrombosis
eCVR: Estimated cardiovascular risk
ESR: Erythrocyte sedimentation rate
ETC: Etanercept
GH: Growth hormone
HDL: High-density lipoprotein
IGF1: Insulin-like growth factor 1
IGF1R: IGF1 receptor
IL: Interleukin
IQR: Inter-quartile range
IRS: Insulin receptor substrate
IS: Ischemic stroke
LDL: Low-density lipoprotein
LE: Lung embolism
MI: Myocardial infarction
MTX: Methotrexate
OR: Odds ratio
RA: Rheumatoid arthritis
RF: Rheumatoid factor
ROC: Receiver operator characteristics
RTX: Rituximab
SD: Standard deviation
SLZ: Sulfasalazine
T2D: Type 2 diabetes mellitus
TC: Total cholesterol
TIA: Transitory ischemic attack
TNF: Tumor necrosis factor
TOCI: Tocilizumab
